# Sulphatation Does Not Appear to Be a Protective Mechanism to Prevent Oxysterol Accumulation in Humans and Mice

**DOI:** 10.1371/journal.pone.0068031

**Published:** 2013-07-03

**Authors:** Jure Acimovic, Anita Lövgren-Sandblom, Maria Olin, Zeina Ali, Maura Heverin, Rebecca Schüle, Ludger Schöls, Björn Fischler, Peter Fickert, Michael Trauner, Ingemar Björkhem

**Affiliations:** 1 Division of Clinical Chemistry, Department of Laboratory Medicine, Karolinska Institutet, Karolinska University Hospital Huddinge, Stockholm, Sweden; 2 Institute of Biochemistry, Faculty of Medicine, University of Ljubljana, Ljubljana, Slovenia; 3 Hertie Institute for Clinical Brain Research and Center of Neurology, University of Tubingen, Tubingen, Germany; 4 Division of Pediatrics (CLINTEC), Karolinska University Hospital, Karolinska Institutet, Stockholm, Sweden; 5 Research Unit for Experimental and Molecular Hepatology, Division of Gastroenterology and Hepatology, Department of Medicine, Medical University, Graz, Austria; 6 Division of Gastroenterology and Hepatology, Department of Internal Medicine III, Medical University of Vienna, Vienna, Austria; Clermont Université, France

## Abstract

24S- and 27-hydroxycholesterol (24OHC and 27OHC) are potent regulators of different biochemical systems *in vitro* and are the major circulating oxysterols. A small fraction of these oxysterols has been reported to be sulphated but there are no detailed studies. We considered the possibility that sulphatation is a protective mechanism preventing accumulation of free oxysterols. Using an accurate assay we found the sulphated fraction of 24OHC and 27OHC in circulation of adults to be less than 15% of total. In two patients with a mutation in CYP7B1 and markedly increased levels of 27OHC the sulphated fraction was 8% and 10% respectively. Infants with severe neonatal cholestasis had however markedly increased sulphate fraction of the above oxysterols. In untreated mice the degree of sulphatation of 24OHC and 27OHC in serum varied between 0 and 16%. Similar degree of sulphatation was found in two mouse models with markedly increased levels of 27OHC and 24OHC respectively. Bile duct ligated mice had higher levels of oxysterols than sham-operated controls but the sulphate fraction was not increased. We conclude that a primary increase in the levels of the oxysterols due to increased synthesis or reduced metabolism in adults and mice does not induce increased sulphatation.

## Introduction

Side-chain oxysterols are oxidized derivatives of cholesterol that are the intermediates of cholesterol excretion pathways [1], [2]. Because of their ability to pass cell membranes and the blood-brain barrier much faster than cholesterol, they are also important as transport forms of cholesterol. In addition, many important roles have been ascribed to oxysterols in connection with cholesterol turnover; i.e. Alzheimer's disease, atherosclerosis, apoptosis, necrosis, inflammation, immunosuppression and development of gallstones [1]–[4]. According to current concepts, oxysterols are physiological regulators in connection with many cholesterol-induced metabolic effects [1], [2]. However, most data supporting such roles are still indirect and there is a marked discrepancy between the documented strong effects of the side-chain oxidized oxysterols under *in vitro* conditions and the very few studies that aim to find their possible physiological importance *in vivo* [1]. Most probably this is a consequence of the un-physiological conditions used for *in vitro* experiments with levels of oxysterols orders of magnitude higher than in the *in vivo* situation. The few transgenic mouse models developed thus far with reduced or increased concentration of oxysterols exhibited surprisingly small changes in cholesterol turnover and homeostasis [5]–[9]. Based on *in vitro* experiments the side-chain oxidized oxysterols 24S-hydroxycholesterol (24OHC) and 27-hydroxycholesterol (27OHC) are supposed to regulate the activity of a number of nuclear liver X receptor (LXR) target genes of importance for cholesterol and lipid homeostasis [10], [11]. 24OHC is the most efficient LXR ligand under *in vitro* conditions and is present in the brain and in the circulation at relatively high concentrations. 27OHC is somewhat less efficient as an activator, although it is the dominating oxysterol in the circulation and in most tissues except the brain. Particularly high levels are found in human macrophages and in atheromas.

We have considered the possibility that sulphatation of the above oxysterols represents a “defence” mechanism that becomes important when the levels of the potent oxysterols increase above a certain level. It has been reported that under normal conditions about 10% of the 24OHC and 27OHC are present as sulphates in human plasma [12], but the methods used have not been validated and have been difficult to reproduce. In the present work, an accurate method for quantification of oxysterol sulphates in serum was developed. The method was used to test the aforementioned hypothesis on several human and mouse serum samples. Serum samples were analysed from healthy volunteers and from two patients with a recessively inherited “pure” hereditary spastic paresis (SPG5) having mutations in the gene coding for the oxysterol 7α-hydroxylase (CYP7B1) [13] and from six children with cholestatic liver disease. In addition we analysed serum samples from two mouse models with markedly increased levels of 24OHC and 27OHC respectively [7], [9]. Furthermore we analysed serum from mice with cholestasis due to a ligation of the common bile duct [14].

## Materials and Methods

### Chemicals

All chemicals and solvents were of highest purity available. 1-Propanol, tetrahydrofuran (THF), methanol, chloroform, cyclohexane, toluene, 2-propanol, and hexane were obtained from E. Merck (Darmstadt, Germany). 2-Hydroxypropyl-β-cyclodextrin (BCD), cholesterol sulphate, 3′-phosphoadenosine 5′-phosphosulphate (PAPS) and sulphur trioxide trimethylamine complex were obtained from Sigma-Aldrich (St. Louis, MO, USA). 24OHC and 27OHC standards were purchased from American Radiolabeled Chemicals (St. Louis, MO, USA) and Avanti Polar Lipids (Alabaster, AL, USA), respectively. 25-hydroxycholesterol (25OHC) was purchased from Steraloids (Newport, RI, USA). Internal standards d6-cholesterol and d6-27OHC were purchased from Medical Isotopes (Pelham, NH, USA) and Avanti Polar Lipids (Alabaster, AL, USA), respectively. d3-24OHC and d3-25OHC were synthesised as described previously [15]. Sulphotransferase family cytosolic 2B member 1 (SULT2B1; EC 2.8.2.2) was purchased from ProSpec (Ness Ziona, Israel) and consisted of SULT2B1 0.5 mg/ml solution containing 20 mM Tris-HCl (pH 7.5) and 10% glycerol.

### Serum Samples from Mice

We analysed serum from 3 female and 4 male C57Bl wild-type mice on normal chow diet.

The 3 female mice were all 44 weeks of age. Two of the males (male 1 and 2) were 8 weeks and two of them (males 3 and 4) were 44 weeks of age.

We analysed serum from 4 females and 2 males with an overexpression of sterol 27-hydroxylase (CYP27A1) causing levels of 27OHC 4–6 fold higher than normal [7]. The genetic background of these mice is C57Bl [7]. The age of the female mice varied between 26 and 39 weeks. The age of the male mice were 38 and 40 weeks.

We analysed serum from 3 female and 5 male mice with an overexpression of sterol 24S-hydroxylase (CYP46A1) and levels of 24OHC 4–6 fold higher than normal [9]. The genetic background of these mice is C57Bl [9]. The age of the female mice varied between 13 and 36 weeks. The age of all the male mice was 17 weeks.

We analysed serum from 3 sham operated and 3 cholecystectomized common bile duct ligated male mice aged 11–12 weeks. This mouse model as well as the surgical procedure has been described in detail [14]. The genetic background of these mice is also C57Bl. The mice were harvested 7 days after surgery.

### Serum Samples from Healthy Volunteers and Patients

We analysed serum from 5 healthy females and 5 healthy males (laboratory staff). The age of the females varied between 31 and 59 years and the age of the males varied between 29 and 71 years.

We analysed serum from two patients with hereditary spastic paresis type SPG5 [13]. Patient 1 is a male, 42 years who has suffered from spastic paresis since the age of 11. Patient 2 is a female, 44 years, who has suffered from spastic paresis since the age of 18.

We analysed serum from 6 cholestatic girls (patients 1–4) and 2 cholestatic boys (patients 5 and 6). All these patients had severe cholestasis at the time of collection of the sample and all of them were later liver transplanted. Patient 1 suffered from the Algaille syndrome whereas all the other suffered from biliary atresia. Patients 1–4 were females and had ages varying from 2 to 7 months of age. The two males (patients 5 and 6) were 6 and 3 months old at the time when the sample was collected.

### Procedure

After thawing, serum samples (50–600 µl) were added to glass vials together with 100 ng of d3-24OHC +100 ng of d6-27OHC internal standards. 2 ml of 1-propanol was added, vortexed for 1 min, ultra-sonificated for 20 min and centrifuged at 5000 g for 5 min to precipitate proteins and recover lipids. The liquid phase was transferred into a new vial, and the procedure was repeated once more with 2 ml of 1-propanol. The protein pellet was mechanically disrupted using glass Pasteur pipette. 1-propanol liquid phases were pooled, equally divided to new vials and evaporated under a stream of argon at 40°C. One aliquot was hydrolysed and the other first solvolysed and then hydrolysed. 1-Propanol procedure was found to be more reliable compared to previously used butanol extraction [12].

### Solvolysis

900 ml of THF, 80 µl of methanol and 10 µl of 2 M sulphuric acid (pH 1) was added to the vial containing dried extract. Argon was flushed through the vial to remove air and the solvolysis was allowed to proceed for 2 h at 45°C. Distilled water (2 ml), and cyclohexane (5 ml) were added, vortexed for 30 s and centrifuged at 5000 g for 5 min. The upper organic phase was transferred into a new vial, and the extraction was repeated once more with 5 ml of cyclohexane. Extracts were pooled and evaporated under a stream of argon in heating blocks at 45°C.

### Alkaline Hydrolysis

1 ml of hydrolysis solution (8 g NaOH dissolved in 20 ml distilled water +180 ml EtOH (99.5%)) was added and shaken for 1 h in a water bath at 65°C. Distilled water (0.5 ml), and cyclohexane (3 ml) were added, vortexed for 30 s and centrifuged at 5000 g for 5 min. The upper organic phase was transferred into a new vial, and the extraction was repeated once more with 3 ml of cyclohexane. Extracts were pooled and evaporated under a stream of argon in heating blocks at 45°C. The residue was dissolved in 1 ml toluene.

### Solid-Phase Extraction

Cholesterol was separated from oxysterols by means of solid-phase extraction. A 100-mg Isolute silica cartridge (Biotage, Uppsala, Sweden) was conditioned with 3 ml hexane. The sample (in 1 ml toluene) was applied to the silica cartridge. After a 1 ml hexane wash, cholesterol was eluted with 8 ml 0.5% 2-propanol in hexane. Oxysterols were then eluted with 5 ml 30% 2-propanol in hexane.

In the oxysterol analyses the cholesterol fraction was discharged. In the analysis of cholesterol no column chromatography was used and the lipid extract was directly used in the recovery experiments.

### Derivatization

TMS reagent (pyridine/hexamethyldisilazane/chloromethylsilane, 3∶2∶1, v/v/v), 100 µl, was added to the dried extracts and the sealed tube was treated at 60°C for 30 min. The solvent and reagents were removed under a stream of argon at 60°C until complete dryness. The residue was dissolved in hexane, in 80 µl for sterols and in 200 µl for cholesterol. The clear hexane phase was transferred into glass vial, suitable for GC/MS injection.

### Gas Chromatography-mass Spectrometry (GC-MS) Analysis

Analysis was performed as described previously [15] with adequate 24OHC, 25OHC and 27OHC standard curve. GC-MS was performed on an Agilent HP 6890N gas chromatograph-Agilent HP 5973 MSD quadropole mass spectrometer using electron impact ionisation mode (Stockholm, Sweden). Two microliters of each sample was injected into the gas chromatograph inlet via autosampler. The injector temperature was held at 250°C throughout the analysis. Injection was performed on a HP-5MS (Scantec Lab AB, Gothenburg, Sweden) capillary column (30 m×0.25 mm, 0.25 µm phase thickness), using helium as carrier gas at a flow rate of 0.8 ml min^−1^. The initial column temperature of 180°C was programmed at a rate of 20°C min^−1^ until reaching 250°C, then raised to 300°C at a rate of 5°C min^−1^ and maintained at this temperature for further 14 min, giving a total runtime of 27.5 min.

The mass spectrometer was operated in the selected ion monitoring mode, and two or four ions were detected at the same time. The ions used for analysis (*m/z*) and typical retention times (minutes) were as follows: d3-24OHC, 416, 15.69; 24OHC, 413, 15.73; d3-25OHC, 134, 15.91; 25OHC, 131, 15.93; d6-27OHC, 462, 16.80, 27OHC, 456, 16.90.

### Synthesis of 24OHC and 25OHC Sulphate

Synthesis of 24OHC sulphate was performed according to Javitt [16] with some modifications. 24OHC was solubilized in 1.67 mM BCD by ultra-sonification. The 1 ml final reaction volume contained 5 µg of SULT2B1, 0.2 mM Tris-HCl (pH 7.5), 0.1% glycerol, 5 mM MgCl_2_, 0.33 mM BCD, 20 µM 24OHC and 40 µM PAPS. Reaction mixture was incubated over-night in water bath at 37°C. The reaction was stopped by addition of 3 ml Folch (chloroform/methanol, 2∶1; v/v). The mixture was vortexed for 1 min and short-spined. The lower chloroform phase containing non-sulphates was transferred into new vial and extraction was repeated once more with 3 ml Folch. The remaining water-methanol contained sulpho-conjugates as confirmed by GC-MS analysis. A bulk volume of chloroform phase and water-methanol phase was treated with and without solvolysis and subjected to GC-MS analysis. The chloroform phase contained identical amounts of 24OHC in the samples treated with and without solvolysis. The water-methanol phase contained no 24OHC in the sample without solvolysis whereas the sample treated with sovolysis contained 24OHC. No further purification was necessary and the material could be used directly in the recovery experiments described in Table 1. 25OHC sulphate was synthesized as described by Ren et. al. [17]. Briefly, a mixture of 25OHC (402 mg, 1 mmol) and sulphur trioxide trimethylamine complex (160 mg, 1 mmol) in 5 ml of chloroform was stirred at 25°C for 7 days. After the solvent was evaporated at reduced pressure, products were purified by high-performance liquid chromatography (HPLC) using silica gel column and methylene chloride and methanol (5%) as mobile phase. The product was purified by reverse phase HPLC using C18 column. The structure of the product was characterised by GC-MS operated in scan mode. The purity was confirmed by GC-MS and HPLC.

**Table 1:** Recovery Experiments using pooled serum sample of healthy volunteers.

Experiment	Measured after solvolysis(no sulphate added) [ng/ml]	Sulphate added [ng/ml]	Expected [ng/ml]	Observed^c^[ng/ml]	Recovery [%]
**Cholesterol**	(x10^6^)				
Sample 1	2.20	0.69^d^	2.89	2.85	98.6
Sample 2	2.17	0.69^d^	2.86	2.89	101.0
Sample 3	2.36	0.74^d^	3.10	3.07	99.0
Sample 4	2.32	0.74^d^	3.06	3.02	98.7
**24OHC** a					
Sample 1	90.1	46.0^e^	136.1	136.3	100.1
Sample 2	75.4	35.4^e^	110.8	119.6	107.9
Sample 3	74.7	35.4^e^	110.1	114.7	104.2
**25OHC** b					
Sample 1	9.7	9.8^f^	19.5	19.2	98.5
Sample 2	9.3	9.8^f^	19.1	18.8	98.4
Sample 3	9.8	9.8^f^	19.6	19.6	100.0

a 24S-hydroxycholesterol,

b 25-hydroxycholesterol,

c Measured after solvolysis with sulphate added,

d cholesterol-sulphate,

e 24OHC-sulphate,

f 25OHC-sulphate.

### Ethical Aspects

Ethical approval was obtained from local ethical committee at Huddinge Hospital with the case number 71/01 to perform “studies on bile acid metabolism in children with cholestatic liver disease”. Informed consents to perform these studies were obtained from the caretakers. This consent was obtained verbally as accepted by the committee. Oxysterols are precursors to bile acids and are thus included in “bile acid metabolism”. The permission was restricted to blood taken in connection with the diagnostic tests. Thus the volume of blood taken in connection with this was greater than was justified for diagnostic reasons. There was no specific documentation for the use of small amounts of blood used for research. Permission to collect blood samples from the healthy volunteers was also obtained from the above ethical committee at Huddinge Hospital with the case number 215/03 to “collect anonymized clinical-chemical analytical results from patients and healthy volunteers to be used for reference purposes or method control”. The consent was obtained both in written form and verbally as accepted by the committee. The study of SPG5 patients is covered by the vote number 690/2011BO1 of the Ethical review board of the Medical Faculty, University of Tübingen. Animal experiments received full approval from The Animal Stockholm Experimentation Ethics Committee (case numbers: S139-10 and S105-11). The experiments with sham operated mice and common bile duct ligated mice were approved by the local Animal Care and Use Committee at the Medical University in Graz, Austria (approval number BMBWK-66.010/0022-BrGT/2005).

### Recovery Calculation

Recovery was calculated by dividing observed concentration (measured concentration of solvolysed human serum with sulphate added) by the expected concentration (sum of measured concentration of solvolysed human serum without addition of sulphate and concentration of sulphate added).

### Statistical Analysis

The R statistical programming language (version 2.15.1) was used for statistical analysis. Two-sided and one-sided independent t-test (with confidence level of 95% and equal variances not assumed) was used to compare percentages and concentrations between sham operated and bile duct ligated mice, respectively.

### Nomenclature

According to a recent paper the preferred nomenclature for 27-hydroxylation and 27-hydroxycholesterol should be (25R)26-hydroxylation and (25R)26-hydroxycholesterol, respectively [18].

## Results and Discussion

### Method

A number of methods for solvolysis has been reported in literature including use of sulphatase from *Helix pomatia* (type-H1), sulphuric acid in ethyl acetate, trifluoroacetic acid in THF [12], [19]–[23]. We tested these methods on cholesterol sulphate using pooled bulk serum sample of healthy volunteers and in our hands the present method utilizing THF and sulphuric acid gave the most reproducible results. When testing the method described above (Materials and Methods) the recovery of cholesterol sulphate was found to be 99.3±0.6% (mean ± standard error of the mean (SEM), see Table 1).

In the recovery experiments with oxysterol sulphates we used synthetic sulphate ester of 24OHC and 25OHC. The yield of the sulphate ester of 24OHC in the synthesis was low, and limited the number of recovery experiments possible to perform. Thus the sulphate ester of 25OHC was synthesized according to a published procedure [17]. It should be emphasized that this compound should behave in the same way as 24OHC sulphate and 27OHC sulphate under the conditions employed.

In the three recovery experiments shown in Table 1 the recovery of 24OHC sulphate varied between 100.1 and 107.9% with mean 104.1±2.3% SEM. Additionally we synthesized the sulphate ester of 25OHC and made three recovery experiments shown in Table 1. The recovery of 25OHC sulphate was found to be 99.0±0.5% (mean ± SEM). Based on the recovery experiments and the low imprecision the method was regarded to be sufficiently accurate for the studies. The accuracy of the method does not, however, allow accurate determination when the degree of sulphatation is less than 5%. It should be emphasized that the focus of the present work was not to define small differences in the degree of sulphatation of oxysterols under different conditions, but to evaluate if such sulphatation is an important mechanism to prevent a primary increase in the level of these oxysterols.

Throughout the present article we will refer to the total level of oxysterol as sum of free, esterified, and sulphated forms of the oxysterol. The GC-MS method coefficient of variation in the range of biological concentrations was found to be 2% for both 24OHC and 27OHC which was calculated by injecting the same oxysterol-containing sample 10 times in a sequence. For this reason all the subsequent analyses were performed by injecting the same sample in sequence three times.

### Analyses of Serum from Healthy Volunteers and Patients

The fraction of sulphated 24OHC in human circulation of healthy volunteers has previously been reported to be 11±1% (mean ± SEM, n = 5) [12]. There is one study in which serum and urine oxysterols had been measured in children with severe cholestatic liver disease. In the latter case only oxysterols present as double conjugates, 3-sulphate and 24-glucoronide, were measured [24].

In the present study five replicas of a human serum pool showed total 24OHC concentration of 68±1 ng/ml with 5±1% of sulphate fraction and 304±7 ng/ml with 8±2% of sulphate fraction of total 27OHC (mean ± SEM). The lower degree of sulphatation in the present study than in the previous may be due to environmental factors.

As shown in Table 2, serum from healthy male and female volunteers of different ages had lower levels of sulphated oxysterols than the above serum pool, ranging from 0 to 2% for 24OHC and 0–5% for 27OHC.

**Table 2:** Serum concentrations of oxysterols conjugated with sulphuric acid in humans.

	Age	24S-hydroxycholesterol^a^		27-hydroxycholesterol^a^	
	[years]	[ng/ml]	Sulphated fraction	[ng/ml]	Sulphated fraction
**Healthy volunteers**					
Female 1	46	81	0%	298	1%
Female 2	57	58	0%	226	1%
Female 3	59	71	0%	229	0%
Female 4	59	66	0%	244	3%
Female 5	31	73	0%	209	3%
Male 1	71	41	2%	163	1%
Male 2	53	56	2%	231	2%
Male 3	34	75	0%	287	5%
Male 4	29	57	2%	175	4%
Male 5	46	58	0%	208	0%
**SPG5 patients**					
Patient 1	42	99	0%	1735	8%
Patient 2	44	80	9%	1570	10%
**Cholestatic children**					
Patient 1	2m	1303	46%	1134	68%
Patient 2	2m	1216	13%	920	59%
Patient 3	6m	1016	26%	1042	88%
Patient 4	7m	772	25%	899	86%
Patient 5	6m	1069	0%	745	74%
Patient 6	2m	1290	6%	3130	94%

a free+esterified+sulphated, m - months.

As also shown in Table 2, two patients with markedly increased levels of 27OHC due to a mutation in CYP7B1 [13] had a relatively low sulphated fraction of this oxysterol, 8% and 10% respectively.

Infants are known to have considerably higher circulatory levels of 24OHC than adults [25]–[27]. The reason for this is believed to be that the relation between the brain and the liver is markedly different from that of adults [27]. The high levels of 24OHC found in the circulation of the present cholestatic infants (Table 2) may thus at least in part be an age effect. In at least half of the cholestatic infants, however, the degree of sulphatation of serum 24OHC was clearly different from that of healthy adult controls. In contrast to the situation with 24OHC, plasma levels of 27OHC have been reported to be lower in healthy and non-cholestatic infants than in healthy adults [25], [26]. In healthy and non-cholestatic infants 1–3 years of age, the mean concentration of 27OHC was reported to be about 60 ng/ml [26]. As shown in Table 2 the levels of 27OHC in the present cholestatic infants were 10–50 fold higher. The degree of sulphatation was very high, varying between 59% and 94%.

We do not know the mechanism behind the very high levels of 27OHC in the present cholestatic infants. Even higher levels of circulating 27OHC than those observed here have been reported in one fatal case of cholestasis in an infant with a mutation in CYP7B1 [28]. 27OHC is a substrate for CYP7B1 and the lack of this hydroxylase may be part of the explanation for the high levels of 27OHC. Since not only 27OHC but also 24OHC was markedly increased in the circulation of this cholestatic infant, and since 24OHC is not a substrate for CYP7B1, the lack of CYP7B1 cannot be the only explanation. The liver failure must have been the results of other factors than the CYP7B1 mutation, although this mutation could have been a contributing factor. From the previous and the present investigations it seems likely that the accumulation of the side-chain oxidized oxysterols in the circulation of cholestatic infants is secondary to the liver failure.

We conclude that a primary increase in the level of 27OHC in adults does not increase the degree of sulphatation. With respect to the high level of sulphatation of oxysterols in the cholestatic infants, we cannot exclude the possibility that this may be an age-related effect. The possibility may also be considered that the increased sulphatation is secondary to increased activity of a sulphotransferase induced by the high levels of bile acids.

### Analyses of Serum from Mouse Models

As shown in Table 3, serum from normal C57Bl control mice on normal diet had a sulphated fraction ranging from 0–7% in case of 24OHC and 0–16% in case of 27OHC. No gender difference could be observed. Serum from mice with markedly elevated levels of 24OHC or 27OHC due to the genetic manipulations had a similar fraction of sulphated oxysterol. It should be pointed out that the two mouse models with increased levels of oxysterols have normal levels of cholesterol [7], [9].

**Table 3:** Serum concentrations of oxysterols conjugated with sulphuric acid in mice.

	Age	24S-hydroxycholesterol^a^		27-hydroxycholesterol^a^	
	[weeks]	[ng/ml]	Sulphated fraction	[ng/ml]	Sulphated fraction
**Normal mice**					
Female 1	44	35	0%	66	6%
Female 2	44	36	4%	62	6%
Female 3	44	32	7%	25	11%
Male 1	8	38	0%	97	0%
Male 2	8	39	0%	77	3%
Male 3	44	31	4%	89	16%
Male 4	44	30	3%	63	0%
**CYP27A1 over-expressed mice**					
Female 1	40	27	0%	413	5%
Female 2	26	31	1%	361	7%
Female 3	44	22	13%	293	6%
Female 4	39	24	6%	368	9%
Male 1	38	20	5%	184	0%
Male 2	40	19	1%	282	2%
**CYP46A1 over-expressed mice**					
Female 1	13	118	0%	52	6%
Female 2	36	164	6%	52	10%
Female 3	29	119	0%	50	5%
Male 1	17	161	0%	90	4%
Male 2	17	135	1%	105	1%
Male 3	17	127	3%	89	6%
Male 4	17	88	0%	86	0%
Male 5	17	118	0%	96	1%

a free+esterified+sulphated.

The high levels of oxysterols and oxysterol sulphate found in infants with cholestasis promped us to investigate a possible effect of cholestasis in a mouse model. We compared mice that had been sham-operated with common bile duct ligated mice [14]. The latter mice get a severe cholestasis with about 60-fold increase in bile acid concentration in serum and about 30-fold increase in alkaline phosphatase [14]. The total levels of the side-chain oxidized oxysterols were significantly higher in the serum of the cholestatic mice than in that from corresponding sham-operated controls (Table 4). The degree of sulphatation was higher in the serum from sham-operated controls than in that from untreated C57Bl mice. Whether this is due to the sham procedure cannot be evaluated. Interestingly, the degree of sulphatation was lower in the cholestatic mice than in the sham operated controls. The possibility must be considered that this is a consequence of the high levels of bile acids and that there is a competition between bile acids and oxysterols for the same sulphating enzyme system.

**Table 4:** Serum concentrations of oxysterols conjugated with sulphuric acid in cholestatic mice.

	24S-hydroxycholesterol^a^		27-hydroxycholesterol^a^	
	[ng/ml]	Sulphated fraction	[ng/ml]	Sulphated fraction
Sham operated	21±1	23±4%	62±6	26±4%
Bile duct ligated	67±19	2±1%	106±14	4±2%
	P = 0.067	P = 0.030	P = 0.035	P = 0.012

a free+esterified+sulphated.

Values are presented as mean ± SEM; n = 3.

The reason for the relatively high degree of sulphatation of oxysterol in the sham-operated controls is difficult to explain. The surgical trauma should be similar in the sham-operated controls as in the bile duct ligated mice. These experiments were however carried out in a laboratory different from that in which all the other experiments were performed and we cannot exclude environmental effects or minor strain differences. We can also not exclude the possibility that the surgical trauma in itself may increase sulphatation but that this effect is compensated for in the bile-duct ligated mice because of the high levels of bile acids.

### Is Sulphatation of Side-chain Oxysterols a “Defence Mechanism”?

There is no evidence for toxic effects of the high oxysterol levels in our two mouse models [7], [9]. In view of the potent effects of 24OHC and 27OHC under *in vitro* condition, there is however, a potential for such effects. If sulphatation is a general “defence mechanism” to avoid potential toxic effects of free side-chain oxidized oxysterols, one would expect the markedly elevated levels of 27OHC or 24OHC in our two moue models to be associated with an increased sulphate fraction. The fraction of the oxysterol pool that was sulphated was however about the same as that of the controls (Table 3). The levels of oxysterols in the circulation of cholestatic mice were also increased without an increase of the sulphated fraction (Table 4). The sulphated fraction of 27OHC in the circulation of the two patients with a mutation in CYP7B1 was similar to or only slightly higher than that of the controls (Table 2). Also this result does not support the concept that sulphatation is a “defence” mechanism. In the cholestatic infants, the degree of sulphatation was clearly increased, in particular in case of 27OHC. We cannot exclude the possibility that this is an age-dependent effect rather than a response to high levels of oxysterols.

According to the results of the present study side-chain oxidized oxysterols are not likely to be primary inducers of increased sulphatation, at least not in adults or in mouse models.
